# Community pharmacists’ views towards implementing a patient self-administered screening tool designed to identify risk of medication-related problems

**DOI:** 10.3389/fphar.2025.1531500

**Published:** 2025-03-26

**Authors:** Mohammed S. Salahudeen, Ahmed Samy Saadeldean, Gregory M. Peterson, Behailu Terefe Tesfaye, Colin M. Curtain

**Affiliations:** ^1^ School of Pharmacy and Pharmacology, College of Health and Medicine, University of Tasmania, Hobart, TAS, Australia; ^2^ School of Pharmacy, Faculty of Health Science, Jimma University, Jimma, Ethiopia

**Keywords:** community pharmacist, medication-related problem, drug-related problem, screening tool, self-administered, medication safety, community pharmacy

## Abstract

**Objective:**

There is limited information regarding community pharmacists’ perspectives on implementing a self-administered screening tool for identifying patients at risk of medication-related problems. This study assessed Australian pharmacists’ views on introducing such a tool within the community pharmacy setting.

**Methods:**

An online cross-sectional survey was conducted among Australian community pharmacists from March to May 2023. The survey collected relevant demographic data and responses on perceived barriers and facilitators to implementing the screening tool. Reliability statistics were computed for the responses on barriers and facilitators, and chi-square or Fisher’s Exact tests were performed to assess their association with demographic variables.

**Results:**

Two hundred thirty-one community pharmacists across Australia were surveyed. Most (78%) reported that medication-related problems are common and expressed support for a patient self-administered screening tool to identify patients at high risk of medication-related problems (88%). Over two-thirds (69%) were willing to allocate time for reviewing patient medications if flagged for medication-related problems. The most frequently anticipated barriers to implementing screening tools were time constraints for pharmacists (63%), staff shortage and limited patient interest (each accounting for 57%). In contrast, effective communication with patients (69%) and patients’ appreciation of pharmacists’ expertise and efforts (67%) were predominantly stated facilitators.

**Conclusion:**

Most community pharmacists were supportive of implementing a patient self-administered screening tool to identify patients at risk of medication-related problems. The study’s findings provide valuable insights for developing medication-related problems screening tools tailored to the Australian community pharmacy setting.

## 1 Introduction

A medication-related problem (MRP) is defined as any event or situation involving medication therapy that either actually or potentially interferes with desired health outcomes (Cipolle et al., 2012). According to the Pharmaceutical Care Network Europe (PCNE), MRP encompasses unnecessary drug therapy, insufficient drug therapy, ineffective drug therapy, adverse drug events, incorrect dosages, and suboptimal adherence (Pharmaceutical Care Network Europe, 2020). These problems are associated with considerable adverse impact on the healthcare system. For instance, in Australia, around 250,000 hospital admissions annually are estimated to be due to MRPs, costing AUD$1.4 billion (Lim et al., 2022). Of these, over two-thirds are potentially preventable (Lim et al., 2022), highlighting the opportunity to reduce this burden. With the ageing population (Australian Institute of Health and Welfare, 2023) and increasing rates of multimorbidity and medicine use, MRPs are likely to become more common in Australia (Nicosia et al., 2019).

Various tools have been developed to identify and reduce the risk of MRPs. For instance, in the United States, Beer’s Criteria was published in 1991 (American Geriatrics Society Beers Criteria^®^ Update Expert Panel, 2023) followed by McLeod’s Criteria from Canada (McLeod et al., 1997). These tools were developed to guide clinicians in reducing the prescribing of inappropriate medications for older adults and patients with specific conditions (McLeod et al., 1997; American Geriatrics Society Beers Criteria^®^ Update Expert Panel, 2023). Some tools and questionnaires are focused on adverse effects or adherence (Butler et al., 2004; Wetzels et al., 2006), while others screen for a wide range of MRPs (Barenholtz Levy, 2003; Paulino et al., 2004; Blalock and Patel, 2005; Gordon et al., 2005; Pammett et al., 2015). One study by Levy et al. developed a tool to be completed solely by the patient independent of healthcare professional involvement (Barenholtz Levy, 2003). The screening tools have been trialled in small-large groups of patients. Furthermore, a systematic review of eleven MRP risk assessment (screening) tools has revealed significant variations in content among these tools (Puumalainen et al., 2019).

Community pharmacists have the potential to play a vital role in recognising patients at risk of MRPs due to their frequent patient interactions, ready accessibility, and expertise in medicines (Pharmacy Guild of Australia, 2021). In Australia, as part of measures to mitigate MRPs, the government funds programs, such as the MedsCheck and Diabetes MedsCheck (DMC), so community pharmacists can assist patients in improving their health and optimising medicine use (Pharmacy Programs Administrators, 2021; Gargya et al., 2022). However, these services do not benefit everyone taking medication, indicating the need for comprehensive tools. There is limited understanding of pharmacists’ perceptions and insights regarding the implementation of patient self-administered tools for identifying MRPs in community pharmacy setting. It is well-documented that community pharmacists often work under significant time constraints, particularly in busy environments (Pharmacy Guild of Australia, 2021). These pressures may discourage pharmacists from adopting additional tools for addressing MRPs.

Given their unique role, community pharmacists can provide valuable insights into the essential features and content required for a screening tool specifically tailored to the Australian setting. Furthermore, they can offer perspectives on potential barriers and facilitators to implementing such a tool within the pharmacy workflow. Hence, our study aimed to evaluate community pharmacists’ views on implementing a patient self-administered screening tool to identify at-risk patients, contributing to a practical approach for improving MRP identification in the community.

## 2 Methods

### 2.1 Study design and setting

This online cross-sectional national survey was conducted in Australia between March and May 2023 and was hosted on the Qualtrics platform (Qualtrics.XM, 2023). The study targeted Australian Health Practitioner Regulation Agency (AHPRA)-registered community pharmacists across Australia.

### 2.2 Sample size and recruitment of pharmacists

According to the Pharmacy Guild of Australia, there were approximately 20,000 pharmacists employed in community pharmacy settings (Pharmacy Guild of Australia, 2021). Based on a 95% confidence interval and a 5% margin of error, an estimated sample size of 377 participants was determined for this survey, based on a sample size calculator provided by Qualtrics (https://www.qualtrics.com/blog/calculating-sample-size/).

To recruit participants, an advertisement with a survey link was shared on Australian pharmacy-focused groups and social media platforms, including Facebook, Twitter, and LinkedIn. An invitation letter, containing both a survey link and a QR code, was also sent to pharmacy organisations, such as the Pharmaceutical Society of Australia (PSA) Tasmanian Branch, PSA Early Career Pharmacist, Consultant Pharmacists Australia (CoPA), Professional Pharmacists Australia, and Pharmacy Daily. Additionally, Raven’s Recruitment (a pharmacist recruitment agency) and Australian Locum Pharmacists Facebook group were contacted to further promote participation in the survey.

### 2.3 Development of the survey and data collection

The survey questionnaire was developed through a structured process that included a literature review, expert opinion, and feedback from community pharmacists. The questionnaire covered key areas, including participant demographics, characteristics of MRPs encountered in community pharmacy, pharmacists' perspectives on the benefits and essential features of a suitable MRP screening tool, and their views on the barriers and facilitators to implementing a brief, patient-administered MRP screening tool in the Australian community pharmacy setting.

To provide context for participants, the survey included a reference screening tool, the ‘Medication Risk Questionnaire’ developed by Barenholtz Levy (Barenholtz Levy, 2003), to assess the risk of MRPs in community pharmacy. Participants were invited to offer suggestions for improvements, evaluate the tool’s feasibility, and provide insights into its integration into the pharmacy workflow (e.g., completion time, applicability).

A pilot test was conducted with five registered pharmacists to assess face and content validity. Based on their feedback, revisions were made to improve clarity and ease of completion. The final version, designed for a 10–15-min completion time, included five sections with Likert scales, closed, and open-ended questions. Section A collected demographic information, Section B addressed MRPs, Section C focused on patient-administered screening tools for identifying MRPs, and Section D explored barriers and facilitators to implementation. Data were collected via Qualtrics between March and May 2023. The full survey questionnaire is provided in Supplementary Table S1.

### 2.4 Outcome measures

The primary outcome measures included community pharmacists’ views and attitudes towards implementing a patient self-administered screening tool designed to identify patients at potential risk of MRPs in community pharmacy. Additionally, they provided perspectives on potential barriers and facilitators for implementing such a tool effectively within the pharmacy workflow.

### 2.5 Data analysis

Survey data were imported into IBM SPSS version 28 for analysis. All responses were reviewed for completeness, and any incomplete entries were excluded from further analysis. Descriptive statistics, including median with range, frequencies, and percentages, were calculated as appropriate. Inferential statistics were conducted using chi-square tests or Fisher’s Exact tests to examine associations on beliefs, barriers, and facilitators for implementing a screening tool. The chi-square test was employed when expected cell counts were ≥5 in at least 80% of cells, with no cell containing a zero. If these conditions were not met, Fisher’s exact test was used.

Items related to barriers and facilitators were assessed on a 5-point Likert scale (5 = strongly agree, 4 = agree, 3 = neutral, 2 = disagree, and 1 = strongly disagree). For the purpose of running chi-square tests or Fisher’s Exact Test and to simplify interpretation, Likert responses were re-categorised as follows: “Agree” (combining “strongly agree” and “agree”) and “Disagree” (combining “neutral,” “disagree,” and “strongly disagree”), assuming the psychological distance between categories was uniform.

The reliability of the barriers and facilitators questions was evaluated using Cronbach’s alpha, with a value above 0.7 considered acceptable. Cronbach’s alpha values for the barriers and facilitators questions were 0.77 and 0.75, respectively, indicating acceptable internal consistency. Additional open-ended responses regarding barriers and facilitators were thematically summarised inductively. All p-values less than 0.05 were considered statistically significant.

We followed the reporting guidelines of the STROBE (Strengthening the Reporting of Observational Studies in Epidemiology) statement for observational studies (Elm et al., 2007).

## 3 Results

### 3.1 Descriptive findings

Among the total survey responses received (n = 256), we included 231 completed responses (90%) in the final analysis. The respondents were predominantly in the age range of 20–39 years (77%). Pharmacists or pharmacists in charge accounted for 78%, and over two-thirds (67%) had practised as a community pharmacist in Australia for less than 10 years (Table 1).

**TABLE 1:** Demographics of the study participants.

Demographic information	Frequency, n (%)
Age	
20–29	73 (31.6%)
30–39	104 (45%)
40–49	37 (16%)
50–59	13 (5.6%)
60 and above	4 (1.7%)
Gender	
Male	128 (55.4%)
Female	98 (42.4%)
Non-binary/third gender	5 (0.02%)
Years of practising as a community pharmacist in Australia	
< 5	57 (24.7%)
5–10	98 (42.4%)
10–15	57 (24.7%)
15–20	12 (5.2%)
> 20	7 (3%)
Principal place of employment (location)	
Urban	132 (57.1%)
Rural	92 (39.8%)
Remote	7 (3%)
Role in the community pharmacy	
Pharmacist	90 (39%)
Pharmacist In Charge	89 (38.5%)
Pharmacist Manager	33 (14.3%)
Pharmacist Owner-operator	19 (8.2%)
Work in a forward dispensing pharmacy	
Yes	174 (75.3%)
No	19 (8.2%)
Unsure	38 (16.4%)
Accredited to do HMR and/or RMMR	
Yes	165 (71.4%)
No	60 (25.9%)
Unsure	6 (2.6%)

HMR, Home Medicine Review; RMMR, Residential Medication Management Review.

Respondents stated that MRPs were common (78%), and most (46%) were moderately severe. The most common issues encountered were improper drug selection (18%), drug interactions (18%) and adverse drug reactions (17%), as shown in Table 2.

**TABLE 2:** Profile of MRPs reported by the respondents.

Reported MRPs profile	Frequency, n (%)
How common are MRPs among patients in your community pharmacy?	
Very common	58 (25.1%)
Common	122 (52.8%)
Not so common	47 (20.3%)
Do not know	4 (1.7%)
Type of MRPs encountered in the last week	
Adverse drug reaction (side effect)	86 (16.8%)
Drug interaction	90 (17.6%)
Improper drug selection	93 (18.2%)
Dose too low	59 (11.5%)
Dose too high	49 (9.6%)
Failure to receive medications	43 (8.4%)
Medication use without an indication	37 (7.2%)
Medication non-adherence	50 (9.8%)
None	4 (0.8%)
The severity of the most significant MRP encountered last week	
Severe (requiring immediate referral to doctor or hospital)	53 (22.9%)
Moderate (requiring counselling and not an urgent referral to a doctor or hospital)	107 (46.3%)
Mild (resolved by counselling)	59 (25.5%)
I did not encounter an MRP last week	12 (5.2%)

MRP, medication-related problem.

Most pharmacists (62.8%) believed a screening tool could help identify patients at risk of MRPs, and 88% perceived that MRP screening tools could be helpful in their community pharmacy. Over two-thirds (69%) said they had time to review patient medications. However, only 55% thought the questions in the reference screening tool (Barenholtz Levy, 2003) would be understood by patients. Respondents were asked for suggestions on whether a particular medication or medication class should be included in an MRP screening tool. They suggested statins, anticoagulants, amiodarone, non-steroidal anti-inflammatory drugs, and medications increasing the risk of falls. Respondents indicated that the MRP screening tool should be completed within 15 min by patients, with or without assistance from staff, to fit within the community pharmacy workflow. Respondents mostly preferred to review a patient’s medication at quiet times during workday by scheduling an appointment with the patient (42.5%), as illustrated in Table 3.

**TABLE 3:** Pharmacists’ Perceptions of self-administered screening tool for identifying patients at greatest risk of MRP.

Pharmacists’ perceptions of patient self-administered MRP screening tool (N=231)	Frequency, n (%)
Do you believe a screening tool would help identify patients at risk of MRPs?	
Yes	145 (62.8%)
No	47 (20.3%)
Unsure	39 (16.9%)
Would a screening tool to identify patients at risk of developing an MRP in your community pharmacy be beneficial?	
Very beneficial	82 (35.5%)
Somewhat beneficial	122 (52.8%)
Not beneficial	15 (6.5%)
Unsure	12 (5.2%)
Are you familiar with any patient self-administered screening tools that help to identify patients at risk of MRPs?	
Yes	54 (23.4%)
No	110 (47.6%)
Unsure	67 (29%)
Do you believe the questions in the sample screening tool shown would be understood by most of your patients?	
Yes	126 (54.5%)
No	59 (25.5%)
Unsure	46 (19.9%)
Do you believe a screening tool would be completed before the patient's medications are ready for collection in the pharmacy?	
Yes	129 (55.8%)
No	64 (27.7%)
Unsure	38 (16.5%)
How long MRP screening tool should be completed to fit within the community pharmacy workflow, Median (IQR)	15 minutes (10–26)
If the screening tool identifies a patient at high risk of potential MRP, would you have the time to review the patient’s medication?	
Yes	160 (69.3%)
No	43 (18.6%)
Unsure	28 (12.1%)
If yes to above response, when you would have the time to review the patient’s medication	
While the patient is waiting	52 (32.5%)
At another quiet time during the workday by scheduling an appointment	68 (42.5%)
Arrange a funded (either government or privately) medication review session with the patient (e.g., MedsCheck)	35 (21.9%)
Unsure	5 (3.1%)
What would you do if the patient had a high risk of potential MRP (as identified by the screening tool)	
Provide information to the patient’s general practitioner or other relevant healthcare providers	118 (30.7%)
Provide information directly to the patient/carer	118 (30.7%)
Make a record of the MRP in the patient’s profile	95 (24.7%)
Record a clinical intervention	47 (12.2%)
Unsure	2 (0.5%)

MRP, medication-related problem.

### 3.2 Barriers and facilitators to implementing patient self-administered screening tool to identify patients at high risk of MRPs

The frequently anticipated barriers to implementing the screening tools were pharmacist time constraints (63%), staff shortage (57%), and lack of patient interest (57%). On the other hand, good communication with the patients (69%), patient appreciation of the pharmacist’s expertise (67%), and support from the employer (65%) were anticipated facilitators for implementing the screening tool, as seen in Table 4. Respondents reported several additional potential barriers and facilitators to implement a self-administered MRP screening tool. These open-ended responses were thematically summarised in Table 5.

**TABLE 4:** Barriers and facilitators to implementing patient self-administered screening tools to identify patients at high risk of MRPs.

Barriers and facilitators	Strongly agree, n (%)	Agree, n (%)	Neutral, n (%)	Disagree, n (%)	Strongly disagree, n (%)
Barriers					
Pharmacist time constraints	54 (23.1%)	94 (40%)	60 (26%)	16 (6.9%)	7 (3%)
Pharmacists’ lack of interest	46 (19.9%)	71 (30.7%)	62 (26.8%)	41 (17.7%)	11 (4.8%)
Lack of employer support	39 (16.9%)	79 (34.2%)	63 (27.3%)	33 (14.3%)	17 (7.4%)
Shortage of staff at work	39 (16.9%)	93 (40.3%)	55 (23.8%)	36 (15.6%)	8 (3.5%)
Lack of appropriate tools to identify MRPs	33 (14.3%)	94 (40.7%)	65 (28.1%)	25 (10.8%)	14 (6.1%)
Insufficient clinical pharmacy training to identify and address MRPs	29 (12.6%)	82 (35.5%)	62 (26.8%)	41 (17.7%)	17 (7.4%)
Insufficient funding/remuneration	47 (20.3%)	78 (33.8%)	65 (28.1%)	28 (12.1%)	13 (5.6%)
Lack of healthcare professionals’ cooperation	37 (16%)	86 (37.2%)	75 (32.5%)	27 (11.7%)	6 (2.6%)
Lack of patients’ interest/time	42 (18.2%)	90 (39%)	55 (23.8%)	34 (14.7%)	10 (4.3%)
Patient concerns over confidentiality	27 (11.7%)	85 (36.8%)	79 (34.20%)	26 (11.3%)	14 (6.1%)
Lack of appreciation from patients	29 (12.6%)	76 (32.9%)	74 (32%)	39 (16.9%)	13 (5.6%)
Space constraints	21 (9.1%)	100 (43.3%)	71 (30.7%)	30 (13%)	9 (3.9%)
Facilitators					
Pharmacists’ time/availability for identifying MRPs	40 (17.3%)	91 (39.4%)	66 (28.6%)	20 (8.7%)	14 (6.1%)
Collaboration with HCPs	43 (18.6%)	105 (45.5%)	55 (23.8%)	23 (10%)	5 (2.2%)
Support from the employer	50 (21.6%)	101 (43.7%)	55 (23.8%)	21 (9.1%)	4 (1.7%)
Support from the pharmacy staff	51 (22.1%)	93 (40.3%)	67 (29%)	16 (6.9%)	4 (1.7%)
Patients’ appreciation of pharmacists’ expertise and efforts	55 (23.8%)	100 (43.3%)	50 (21.6%)	19 (8.2%)	7 (3%)
Provision of medicines safety education and awareness to patients	44 (19%)	96 (41.6%)	68 (29.4%)	19 (8.2%)	4 (1.7%)
Screening tool with fewer questions	40 (17.3%)	91 (39.4%)	71 (30.7%)	21 (9.1%)	8 (3.5%)
Good communication with the patients	64 (27.7%)	96 (41.6%)	49 (21.2%)	19 (8.2%)	3 (1.3%)

MRP, medication-related problem; HCP, healthcare practitioner.

**TABLE 5:** Additional barriers and facilitators reported by pharmacists for implementing self-administered screening tools to identify patients at high risk for MRPs.

Barriers
1. Patient-related challenges
• Patients may be unwilling to do screening tool questionnaire
• Patients do not have enough time
• Language barrier where the tool is in English
• Cultural differences
• Lack of patient knowledge about the role of pharmacists and their role in medication management and medicine safety
• Patients’ perception that the pharmacist is ”too busy“
• Many patients don't see the point of doing this
• Customers care only about quick medicine and whatever they want. If you ask too much, they will abuse pharmacists at retail pharmacies
• Some patients assume they know it all
2. Operational and workflow issues
• Incorporating into workflow processes to then tangibly deliver something clinically beneficial - without a remuneration model supporting its use
• Lack of actual mechanics or procedural details on how to implement
• Screening tool that is not comprehensive and easy to use
• HMR type review is too expensive and time-consuming
• No-Australian specific tools
3. External stakeholder barriers
• Fear of rejection or lack of GP interest/reluctance after receiving a referral from a pharmacist
• Prescribers’ time constraints
• Owners only care about profit
4. Systemic and resource limitations
• Drug unavailability
• Patient support
Facilitators
1. Patient empowerment and awareness
• Empowering patients
• Enhance rural pharmacy services
• Increasing awareness to patients (media campaigns and other means)
2. Pharmacist training and collaboration
• Training for pharmacists
• Collaboration with GPs
3. Tool design and utility
• The comprehensiveness of medicine

### 3.3 Association between barriers and demographics of the respondents’

Several demographic factors were significantly associated with the barriers to implementing a self-administered MRP screening tool (Table 6). Eight out of the 12 barriers to the implementation of the MRP screening tool showed a statistically significant association with various demographics of the participants. For instance, pharmacist time constraints were linked to years of practice (p = 0.023), working in forward-dispensing pharmacies (p = 0.009), and accreditation to conduct Home Medicine Reviews (HMR) and/or Residential Medication Management Reviews (RMMR) (p = 0.042). Similarly, a lack of pharmacist interest was associated with the principal place of employment (p = 0.039). Other notable findings included patient confidentiality concerns being associated with years of practice (p = 0.035) and pharmacist’s role (p = 0.002), while funding and remuneration challenges were tied to age group (p = 0.019), years of practice (p = 0.033), and place of employment (p = 0.060). Conversely, no significant associations were observed for barriers such as lack of employer support, patient interest or time, staff shortages, or healthcare professionals’ cooperation. The details are presented in Table 6.

**TABLE 6:** The association of pharmacists’ perception of the anticipated barriers to implementing self-administered screening tools to identify patients at high risk of MRPs with demographics.

Demographics	Anticipated barriers															
	Pharmacist time constraints		Pharmacists' lack of interest		Lack of appropriate tools to identify MRPs		Insufficient clinical pharmacy training to identify and address MRPs		Insufficient funding or remuneration		Patient concerns over confidentiality		Lack of appreciation from patients		Space constraints for keeping infrastructure to use screening tool	
	n (%)	p-value	n (%)	p-value	n (%)	p-value	n (%)	p-value	n (%)	p-value	n (%)	p-value	n (%)	p-value	n (%)	p-value
Age category																
20–29	45 (30.4)	0.320	36 (30.8)	0.261	40 (31.5)	0.929	37 (33.3)	0.171	28 (22.4)	**0.019**	40 (35.7)	0.135	31 (29.5)	0.375	39 (32.2)	**0.006**
30–39	64 (43.2)		51 (43.6)		57 (44.9)		49 (44.1)		65 (52)		49 (43.8)		51 (48.6)		53 (43.8)	
40–49	24 (16.2)		24 (20.5)		19 (14.9)		21 (18.9)		21 (16.8)		13 (11.6)		18 (17.1)		17 (14.1)	
50–59	11 (7.4)		4 (3.4)		8 (6.3)		4 (3.6)		8 (6.4)		9 (8)		5 (4.8)		12 (9.9)	
60 and above	4 (2.7)		2 (1.7)		3 (2.4)		0		3 (2.4)		1 (0.9)		0		0	
Years practising as a community pharmacist (in year)																
<5	34 (23)	**0.023**	27 (23.1)	0.093	27 (21.3)	0.261	27 (24.3)	0.468	28 (22.4)	**0.033**	36 (32.1)	**0.035**	27 (25.7)	0.298	39 (32.2)	**0.034**
5–10	65 (4.9)		48 (41)		57 (44.9)		49 (44.1)		47 (37.6)		44 (39.3)		43 (41)		44 (36.4)	
10–15	31 (21)		36 (30.8)		29 (22.8)		29 (26.1)		36 (28.8)		21 (18.7)		26 (24.8)		26 (21.5)	
15–20	11 (7.4)		5 (4.3)		8 (6.3)		5 (4.5)		7 (5.6)		8 (7.1)		8 (7.6)		8 (6.6)	
>20	7 (4.7)		1 (0.8)		6 (4.7)		1 (0.9)		7 (5.6)		3 (2.7)		1 (0.9)		4 (3.3)	
Gender																
Male	85 (57.4)	0.424	67 (57.3)	0.715	72 (56.7)	0.320	63 (56.8)	0.931	71 (56.8)	0.322	64 (57.1)	0.496	54 (51.4)	0.454	73 (60.3)	**0.023**
Female	61 (41.2)		47 (40.2)		54 (42.2)		46 (41.4)		53 (42.4)		47 (42)		48 (45.7)		48 (39.7)	
Non-binary/third gender	2 (1.4)		3 (2.6)		1 (0.8)		2 (1.8)		1 (0.8)		1 (0.9)		3 (2.9)		0	
Employment location																
Urban	91 (61.5)	0.151	58 (49.6)	**0.039**	80 (63)	0.137	64 (51.2)	0.060	76 (61.8)	0.142	70 (62.5)	0.079	51 (48.6)	**0.019**	74 (61.2)	0.340
Rural	54 (36.5)		56 (47.9)		44 (34.6)		55 (44)		42 (34.1)		41 (36.6)		52 (49.5)		43 (35.5)	
Remote	3 (2)		3 (2.6)		3 (2.4)		6(4.8)		5 (4.1)		1 (0.9)		2 (1.9)		4 (3.3)	
Role in the pharmacy																
Pharmacist	63 (42.6)	0.245	37 (31.6)	**0.002**	41 (32.8)	0.183	53 (41.7)	**0.021**	41 (32.8)	0.183	53 (47.3)	**0.002**	38 (36.2)	0.177	48 (39.7)	0.390
Pharmacist in charge	53 (35.5)		58 (49.6)		51 (40.8)		49 (38.6)		51 (40.8)		45 (40.2)		48 (45.7)		51 (42.1)	
Pharmacy manager	18 (12.2)		17 (14.5)		21 (16.8)		19 (15)		51 (16.8)		11 (9.8)		13 (12.4)		14 (11.6)	
Pharmacy owner	14 (9.5)		5 (4.3)		12 (9.9)		6 (4.7)		12 (9.6)		3 (2.7)		6 (5.7)		8 (6.6)	
Work in a forward dispensing pharmacy																
Yes	119 (80.4)	**0.009**	94 (80.3)	**0.024**	101 (79.5)	0.197	87 (78.4)	0.585	97 (77.6)	0.221	92 (82.1)	0.061	78 (74.3)	0.806	99 (81.8)	**0.018**
No	13 (8.8)		4 (3.4)		10 (7.9)		8 (7.2)		12 (9.6)		6 (5.4)		8 (7.6)		10 (8.3)	
Unsure	16 (10.8)		19 (16.2)		16 (12.6)		16 (14.4)		16 (12.8)		14 (12.5)		19 (18.1)		12 (9.9)	
HMR and/or RMMR accredited																
Yes	113 (76.3)	**0.042**	90 (76.9)	0.071	97 (76.4)	**0.009**	86 (77.5)	0.126	89 (71.2)	0.584	86 (76.8)	0.158	73 (69.5)	0.560	87 (71.9)	0.983
No	33 (22.3)		23 (19.7)		30 (23.6)		23 (20.7)		34 (27.2)		23 (20.5)		28 (26.7)		31 (25.6)	
Unsure	2 (1.4)		4 (3.4)		0		2 (1.8)		2 (1.6)		3 (2.7)		4 (3.8)		3 (2.5)	

MRP, medication-related problem; HMR, Home Medicine Reviews; RMMR, Residential Medication Management Reviews. Note: all the frequencies (n) and percentages (%) in the table represents the Likert response “Agree” (combining “strongly agree” and “agree”). Chi-square or Fisher’s Exact Test was employed to assess the existence of association between the variables. The bold values indicate variables with a statistically significant association.

### 3.4 Association between facilitators and demographics of the respondents’

The perception of pharmacists regarding facilitators for implementing patient self-administered screening tools showed notable associations with specific demographic variables. Collaboration with healthcare professionals was significantly associated with pharmacists’ age category (p = 0.012), gender (p = 0.008), and accreditation to conduct HMR and/or RMMR (p = 0.042). Furthermore, the provision of education and awareness to patients about medicine safety was linked to working in a forward dispensing pharmacy (p = 0.026). The perception of screening tools with fewer questions was associated with pharmacists’ gender (p = 0.019). Lastly, good communication with patients was strongly associated with working in a forward dispensing pharmacy (p = 0.001). These key associations are detailed in Table 7.

**TABLE 7:** The association of pharmacists’ perception of the facilitators to implementing patient self-administered screening tools to identify patients at high risk of MRPs with demographics.

Demographics	Anticipated facilitators											
	Collaboration with healthcare professionals		Support from the pharmacy staff		Patients’ appreciation of pharmacists’ expertise and efforts		Provision of education and awareness to patients about medicines safety		Screening tool with fewer questions		Good communication with the patients	
	n (%)	p-value	n (%)	p-value	n (%)	p-value	n (%)	p-value	n (%)	p-value	n (%)	p-value
Age category												
20–29	41 (27.7)	**0.012**	47 (32.6)	0.929	48 (31)	0.445	46 (32.9)	0.883	35 (26.7)	0.382	46 (28.7)	0.221
30–39	72 (48.6)		65 (45.1)		69 (44.5)		60 (42.9)		63 (48.1)		70 (43.8)	
40–49	19 (12.8)		21 (14.6)		23 (14.8)		22 (15.7)		21 (16)		30 (18.8)	
50–59	12 (8.1)		8 (5.6)		11 (7.1)		9 (6.4)		9 (6.9)		10 (6.2)	
60 and above	4 (2.7)		3 (2.1)		4 (2.6)		3 (2.1)		3 (2.3)		4 (2.5)	
Gender												
Male	81 (54.7)	**0.008**	80 (55.6)	1.000	90 (58.1)	**0.006**	75 (536)	0.824	71 (54.2)	**0.019**	85 (53.1)	0.129
Female	67 (45.3)		61 (42.4)		65 (41.9)		62 (44.3)		60 (45.8)		73 (45.6)	
Non-binary/third gender	0		3 (2.1)		0		3 (2.1)		0		2 (1.3)	
Work in a forward dispensing pharmacy												
Yes	118 (79.7)	0.059	113 (78.5)	**0.009**	128 (82.6)	**<0.001**	110 (78.6)	**0.026**	106 (80.9)	0.050	126 (78.8)	**0.001**
No	12 (8.1)		15 (10.4)		12 (7.7)		14 (10)		10 (7.6)		17 (10.6)	
Unsure	18 (2)		16 (11.1)		15 (9.7)		16 (11.4)		15 (11.5)		17 (10.6)	
HMR and/or RMMR accredited												
Yes	113 (76.4)	**0.042**	108 (75)	0.252	120 (77.4)	**0.012**	103 (73.6)	0.594	97 (74)	0.623	115 (71.9)	0.764
No	33 (22.3)		33 (22.9)		31 (20)		33 (23.6)		31 (23.7)		40 (25)	
Unsure	2 (1.3)		3 (2.1)		4 (2.6)		4 (2.9)		3 (2.3)		5 (3.1)	

MRP, medication-related problem; HMR, Home Medicine Reviews; RMMR, Residential Medication Management Reviews. Note: all the frequencies (n) and percentages (%) in the table represents the Likert response “Agree” (combining “strongly agree” and “agree”). Chi-square or Fisher’s Exact Test was employed to assess the existence of association between the variables. The bold values indicate variables with a statistically significant association.

## 4 Discussion

This survey assessed community pharmacists’ views on implementing a self-administered screening tool for identifying patients at high risk of MRPs within community pharmacy setting. In Australia, although there have been attempts to develop MRP screening tools in various non-community pharmacy setting environments (Taylor et al., 2022; Wembridge et al., 2023) and to integrate electronic documentation of MRPs in community pharmacy setting (Williams et al., 2011), evidence regarding community pharmacists’ views on a patient self-administered, community pharmacist-based MRP screening tool is limited. Our study found that most pharmacists believe MRPs are common and they were open to implementing a self-administered screening tool to identify patients at high risk for MRPs. The majority of pharmacists identified time constraints as the primary barrier to implementing the screening tool, followed by staff shortages. Conversely, about two-thirds of the pharmacists felt that effective communication with patients and recognition of the pharmacist’s expertise could facilitate the implementation of the tool. Several anticipated barriers (e.g., pharmacist time constraints, insufficient funding or remuneration, and inadequate clinical pharmacy training to identify and address MRPs) and facilitators (e.g., support from pharmacy staff and effective communication with patients) were found to have significant associations with participants’ demographics, such as their principal place of employment. These findings align with existing literature, which highlights that pharmacists often dedicate substantial time to dispensing, supply, and management activities, leaving limited capacity to provide additional professional services (Lounsbery et al., 2009; Karia et al., 2022; Ali et al., 2023).

Previous studies have shown that MRPs are prevalent in Australian community pharmacy (Williams et al., 2011; Collins et al., 2023). This finding aligns with the responses from pharmacists in our study, which highlights the necessity for implementing measures to prevent or address MRPs within these settings. This is also strengthened by the fact that most pharmacists agreed on the need for screening tools to identify patients at high risk of MRPs in their settings. However, a significant portion of pharmacists reported a lack of familiarity with any MRP screening tools. Among those who were familiar, many mentioned the STOPP/START criteria (O’Mahony et al., 2023) and Beer’s criteria (American Geriatrics Society Beers Criteria^®^ Update Expert Panel, 2023). Nonetheless, these criteria are not comprehensive for all age groups and were not designed to serve as patient screening tools in community pharmacy setting (Milton et al., 2008; Lim et al., 2022).

The willingness of community pharmacists to provide extended services may be influenced by several factors, including time constraints. In a study conducted by Jarab et al., 59% of community pharmacists’ indicated their willingness to provide extended community pharmacy services, such as screening for MRPs (Jarab et al., 2024). This percentage is lower than the proportion of community pharmacists’ willing to provide such services in our study. In our study, a significant portion of the surveyed community pharmacists (71%) were accredited to provide HMR and/or RMMR. Consequently, these respondents are likely to express interest in using MRP screening tools to enhance patient outcomes. Most of the surveyed participants were also pharmacists or pharmacists-in-charge, who are typically responsible for the day-to-day operations of community pharmacies, making them well-positioned to recognise the advantages of the MRP screening tool. In addition to the pressing burden of MRPs in community pharmacy, the willingness to implement a screening tool for identifying patients at high risk for MRPs also indicates the presence of need.

Our findings indicated that pharmacists’ time constraints are a significant anticipated barrier to implementing a patient self-administered MRP screening tool. This issue has also been highlighted in similar studies (Cardwell et al., 2018; Hamadouk et al., 2023; Hogervorst et al., 2024). The pharmacists’ preference to review patients’ medications at quieter times during workday by scheduling an appointment with patients, might also reflect the time limitations they face in their daily activities. To address this, we suggest enhancing the efficiency of the MRP screening tool and minimising the time required for the screening process.

Our study also identified lack of remuneration for performing MRP screening activities as another major anticipated barrier to the implementation of MRP screening tool. Integrating the MRP screening task into government-funded services, such as MedsChecks and DMC (Pharmacy Programs Administrators, 2021), HMR, and RMMR (Pharmacy Programs Administrators, 2022), or facilitating service payment by patients, could help for the implementation of the screening tool in everyday practice and ensure its sustainability.

Previous studies in community pharmacy settings (Cardwell et al., 2018; Hamadouk et al., 2023; Hogervorst et al., 2024), identified barriers not mentioned in our research, such as the prioritisation of other clinical tasks, difficulty accessing patient clinical information, and a perceived disconnect from the primary healthcare team (Cardwell et al., 2018). In contrast, our study found several new patient-related barriers, including concerns about confidentiality, a lack of patient appreciation, cultural differences, language barriers (particularly when the tool is in English), patients’ reluctance to engage in MRP screening, and their preference to quickly collect prescriptions rather than engage further.

Effective communication is widely recognised as crucial for improving pharmacy services (de Oliveira and Shoemaker, 2006). Our findings underscore this importance, suggesting that training pharmacists to use MRP screening tools should also include communication and confidence-building components. While previous research noted different facilitators [e.g., perceived professional roles, clinical outcomes, and peer influence (Cardwell et al., 2018)], our study highlights additional ones, such as strengthening rural pharmacy services and using screening tools with fewer questions.

In forward dispensing pharmacies, where there is no physical barrier between pharmacist and patient, pharmacists can counsel patients immediately as they approach the dispensary (Berbatis et al., 2007). Although this model can enhance patient interaction, it may also increase workload, which could explain the strong association we observed between time constraints and forward dispensing practices (p = 0.009). Furthermore, owners and managers in some community pharmacies may emphasise efficiency over extended patient care, potentially dampening staff motivation to adopt additional interventions (Resnik et al., 2000). This may clarify why pharmacists’ roles were strongly associated with perceptions of patient disinterest (p = 0.002).

Location also appears to influence perceptions. Pharmacists in metropolitan areas often face heavier patient flow, leading to workload pressures that may deter them from adopting additional professional tasks (Kanaani et al., 2023). This aligns with the moderate association noted between pharmacists’ lack of interest and their employment location (p = 0.039). Alternatively, when sufficient staff support is available, particularly in forward dispensing pharmacies, pharmacists can conduct activities like MRP screening more effectively (p = 0.009).

### 4.1 Strengths, limitations and future research

With MRPs posing a significant public health issue, this study addresses an important gap in understanding community pharmacists' roles in early identification of high-risk patients. Our findings offer insights into the attitudes and perspectives of pharmacists who are particularly interested in reducing MRPs within the community setting. The survey may not fully represent all community pharmacists across Australia due to convenience sampling methods or overrepresentation from certain regions, demographic groups, or practice settings (e.g., urban vs. rural areas). Additionally, because the survey was widely advertised across Australia without precise tracking, we were unable to determine an exact response rate. Moving forward, incorporating patient feedback will be essential in determining the feasibility and acceptability of these tools. Exploring artificial intelligence (AI) could further enhance efficiency, as indicated in recent literature (Chalasani et al., 2023; Alsanosi and Padmanabhan, 2024; Graafsma et al., 2024; Maleki Varnosfaderani and Forouzanfar, 2024). Finally, addressing staff shortages by hiring or delegating certain responsibilities to pharmacy technicians may aid in the successful implementation of these screening tools (Michel et al., 2023).

## 5 Conclusion

Despite their demanding schedules, Australian community pharmacists are open to incorporating a patient-self-administered MRP screening tool into their daily routines within the community pharmacy environment. They demonstrate a strong awareness of the prevalence of MRPs in the community and value the recognition they receive from patients for their dedication and expertise. Additionally, effective communication with patients, patient appreciation of pharmacists' expertise, and employer support serves as key facilitators in this process. The study's findings provide valuable insights for developing MRP screening tools tailored to the Australian community pharmacy setting, ensuring they are both feasible and effectively integrated into pharmacists' workflows. Future research should explore implementation strategies and assess the impact of such tools on patient outcomes and pharmacy practice.

## Data Availability

The raw data supporting the conclusions of this article will be made available by the authors, without undue reservation.

## Abbreviations

DMC, Diabetes MedsCheck; HMR, Home Medicine Review; MRP, Medication-Related Problem; PCNE, Pharmaceutical Care Network Europe; PIC, Pharmacists In Charge; RMMR, Residential Medication Management Review; STOPP/START, Screening Tool of Older Persons’ Prescriptions/Screening Tool to Alert to Right Treatment.
