# Supplementary material for: Community pharmacists’ views towards implementing a patient self-administered screening tool designed to identify risk of medication-related problems
Source: Front Pharmacol. 2025 Mar 26;16:1531500. doi: 10.3389/fphar.2025.1531500 (PMC11979211; doi:10.3389/fphar.2025.1531500)
Supplement: Supplementary file 1 [file DataSheet1.pdf]

Supplementary Table 1. Online Survey Questionnaire

Section A - Demographics:

1. What is your age?

- 20-29 years old
- 30-39 years old
- 40-49 years old
- 50-59 years old
- 60 years or older

2. How many years have you been practising as a community pharmacist in Australia?

- < 5
- 5-10
- 10-20
- >20

3. What is your gender?

- Male
- Female
- Non-binary
- Prefer not to answer

4. Where do you reside?

- South Australia
- New South Wales
- Victoria
- Queensland
- Western Australia
- Tasmania
- Northern Territory
- Australian Capital Territory

5. What is your principal employment location?

- Urban
- Rural/regional
- Remote

6. What is your current role in the community pharmacy? Please select the best answer that applies to you.

- Pharmacist
- Pharmacist In Charge (PIC)

- Pharmacist Manager
- Pharmacist Owner-operator

7. Do you work in a forward dispensing pharmacy (*a pharmacy model that allows pharmacists to speak directly with patients and hands out every dispensed prescription to the patient in-person*)?

   - Yes
   - No
   - Unsure

8. How often do you provide professional pharmacy services aiming at reducing medication-related problems and improving the quality use of medicines, such as MedsCheck, Diabetes MedsCheck or other professional services?

9. Are you accredited to do Home Medicine Reviews (HMR) and/or Residential Medication Management Reviews (RMMR)

   - Yes
   - No

Section B - Medication Related Problems (MRPs)

1. In your opinion, how prevalent are MRPs among patients in your community pharmacy?

   - Very prevalent
   - Prevalent
   - Not so prevalent
   - Do not know

2. Over the last week, what was the most notable MRP that you encountered?

   - Severe (*requiring immediate referral to doctor or hospital*)
   - Moderate (*requiring counselling and not an urgent referral to a doctor or hospital*)
   - Mild (*resolved by counselling*)
   - None

3. Would a patient self-administered screening tool to identify patients at greatest risk of developing an MRP in your community pharmacy **be beneficial**?

   - Very beneficial
   - Somewhat beneficial
   - Not beneficial

- Unsure

<u>Section - About patient-administered screening tools to identify MRP.</u>

Are you familiar with any patient self-administered screening tools that help to identify patients at risk of MRPs?

- Yes
- No
- Unsure

If yes, please state which one(s): _______________

Please provide your opinion on the following self-administered screening tool for assessing the risk of medication-related problems in a community pharmacy setting.

Screening Tool A (Medication Risk Questionnaire):

Developed by Hedva Barenholtz Levy. If you would like to view the full study, please follow the link: https://pubmed.ncbi.nlm.nih.gov/12841804/

Ten Questions on the Medication-Risk Questionnaire

1. Do you currently take 5 or more medications?
2. Do you take 12 or more medication doses each day?
3. Do you take any of the following medications: carbamazepine, lithium, phenytoin, quinidine, warfarin, digoxin, phenobarbital, procainamide, theophylline?
4. Does more than 1 physician prescribe medications for you on a regular basis?
5. Are you currently taking medications for 3 or more medical problems?
6. Do you get your prescriptions filled at more than 1 pharmacy?
7. Does someone else bring any of your medications to your home for you?
8. Is it difficult for you to follow your medication regimen or do you sometimes choose not to?
9. Have your medications or the instructions on how to take them been changed 4 or more times in the past year?
10. Of all your medications, is there any particular medicine for which you do not know the reason for taking it?

1) Do you have any suggestions to improve questionnaires for a screening tool to help identify MRPs? _________

2) Do you believe the literacy level in the above screening tool would be understood by most of your patients?
   - Yes
   - No
   - Unsure

3) Do you believe in most circumstances that a screening tool would be completed before the patient's medications are ready for collection after dispensing?
   - Yes
   - No
   - Unsure

4) Do you believe a screening tool would help identify patients at risk of MRPs?
   - Yes
   - No
   - Unsure

5) Do you believe a screening tool would work in your pharmacy?
   - Yes
   - No
   - Unsure

6) How long do you think an MRP screening tool should approximately take to complete by patients, with or without assistance from staff, to fit within your community pharmacy workflow (in minutes)? ___

7) Assume a screening tool to identify patients at risk of MRP has been introduced into your pharmacy.
   a) If the screening tool identifies a patient at high risk of potential MRP, would you have the time to review the patient's medication?
      - Yes
      - No
      - Unsure

   b) If you answered **Yes** to the above question, when would you have the time to review the patient's medication?
      - While the patient is waiting

- At another quiet time during the workday by scheduling an appointment
- Arrange a funded (either government or privately) medication review session with the patient (e.g., MedsCheck)
- Unsure

c) What would you do if the patient had a high risk of potential MRP (*as identified by the screening tool*) that needs to be addressed timely? (Tick all that apply)

- Provide information to the patient's general practitioner or other relevant healthcare providers
- Provide information directly to the patient/carer
- Make a record of the MRP in the patient's profile
- Record a clinical intervention
- Unsure

Section D - Barriers & Facilitators

To what extent do you agree or disagree with the following factors considered **barriers** to adopting a patient self-administered MRP screening tool in your community pharmacy?

**Potential barriers**	**Strongly agree**	**Agree**	**Neutral**	**Disagree**	**Strongly disagree**
Pharmacist time constraints					
Pharmacists' lack of interest					
Lack of employer support					
Shortage of staff at work					
Lack of appropriate tools to identify MRPs					
Insufficient clinical pharmacy training to identify and address MRPs					
Insufficient funding/remuneration					
Lack of health care professionals' cooperation e.g., GPs					
Lack of patients' interest/time					
Patient concerns over confidentiality					
Lack of appreciation from patients					
Lack of patients' awareness about screening tool					

Prioritisation of other pharmacy activities					
COVID-19 restrictions					
Lack of communication skills to engage or convince patients					
Space constraints for keeping a screening tool (e.g., iPad stand)					

Any other potential barriers you would like to mention? ____________________

To what extent do you agree or disagree with the following factors considered **facilitators** to adopting a patient self-administered MRP screening tool in your community pharmacy?

**Potential facilitators**	**Strongly agree**	**Agree**	**Neutral**	**Disagree**	**Strongly disagree**
Saves pharmacists time identifying MRPs					
Improved collaboration with healthcare professionals, e.g., GPs					
Support from the employer					
Support from the pharmacy staff					
Patients' appreciation of pharmacists' expertise and efforts (feel valued)					
Provision of education and awareness to patients about medicines safety					
Screening tool with fewer questions					
Good communication with the patients					

Any other potential facilitators you would like to mention: ________________________
